# Addressing preference heterogeneity in public health policy by combining Cluster Analysis and Multi-Criteria Decision Analysis: Proof of Method

**DOI:** 10.1186/s13561-015-0048-4

**Published:** 2015-05-14

**Authors:** Mette Kjer Kaltoft, Robin Turner, Michelle Cunich, Glenn Salkeld, Jesper Bo Nielsen, Jack Dowie

**Affiliations:** 1 Research Unit for General Practice, Department of Public Health University of Southern Denmark, J.B. Winsløws Vej 9 B, 5000 Odense C, Denmark; 2 School of Public Health and Community Medicine, University of New South Wales, Sydney, NSW 2052 Australia; 3 NHMRC Clinical Trials Centre, Sydney Medical School, Charles Perkins Centre, Johns Hopkins Drive, Camperdown, NSW 2050 Australia; 4 Faculty of Medicine, School of Public Health University of Sydney, Edward Ford Building (A27), Sydney, NSW 2006 Australia; 5 Faculty of Public Health and Policy, London School of Hygiene and Tropical Medicine, 15-17 Tavistock Place, London, WC1H 9SH UK

**Keywords:** Cluster analysis, Multi-criteria decision analysis, Preference subgroups, Heterogeneity

## Abstract

The use of subgroups based on biological-clinical and socio-demographic variables to deal with population heterogeneity is well-established in public policy. The use of subgroups based on preferences is rare, except when religion based, and controversial. *If* it were decided to treat subgroup preferences as valid determinants of public policy, a transparent analytical procedure is needed. In this proof of method study we show how public preferences *could* be incorporated into policy decisions in a way that respects both the multi-criterial nature of those decisions, and the heterogeneity of the population in relation to the importance assigned to relevant criteria. It involves combining Cluster Analysis (CA), to generate the subgroup sets of preferences, with Multi-Criteria Decision Analysis (MCDA), to provide the policy framework into which the clustered preferences are entered. We employ three techniques of CA to demonstrate that not only do different techniques produce different clusters, but that choosing among techniques (as well as developing the MCDA structure) is an important task to be undertaken in implementing the approach outlined in any specific policy context. Data for the illustrative, not substantive, application are from a Randomized Controlled Trial of online decision aids for Australian men aged 40-69 years considering Prostate-specific Antigen testing for prostate cancer.

We show that such analyses can provide policy-makers with insights into the criterion-specific needs of different subgroups. Implementing CA and MCDA in combination to assist in the development of policies on important health and community issues such as drug coverage, reimbursement, and screening programs, poses major challenges -conceptual, methodological, ethical-political, and practical - but most are exposed by the techniques, not created by them.

**Electronic supplementary material:**

The online version of this article (doi:10.1186/s13561-015-0048-4) contains supplementary material, which is available to authorized users.

## Background

Most health care systems are currently under pressure to reconcile the need to deliver services more efficiently and provide more personalised health care. There are a number of reasons for this pressure, including rapid technological advances in medicine and communications, aging populations, and economic crises. A key issue is how population heterogeneity should be respected in policy decisions about health and community issues such as drug coverage, reimbursement and screening. If fully individualised public health care policies are impossible and treating everyone as ‘average’ is unsatisfactory, then what subgroupings represent the optimal compromise, and how are they to be incorporated into public policy?

The case for using subgroups based on *biological-clinical and socio-demographic variables* to address heterogeneity is well-established in *effectiveness* research, with the main issues being the statistical and clinical/policy significance of such analyses. Subgrouping in *cost-effectiveness* is the focus of ongoing debate, largely concerning the use of particular variables for subgrouping rather than the case for subgrouping in principle. Subgrouping based on age and clinical history is widely employed in analyses for organisations determining cost-effectiveness within specific settings, such as NICE in England and Wales [1]. What remains controversial is the use of subgrouping on the basis of individual *preferences or values*, moving beyond clustering based on such concepts as patient satisfaction [2] or healthcare decision making competencies and motivations [3].

The controversy is subdued in the case of most effectiveness research, where it is accepted that key determinants of effectiveness, especially treatment adherence, may be influenced by individual preferences independent of the person’s biological-clinical or socio-demographic characteristics [4]. Little concern has been shown when the suggestion is made that clustered results from individual decision analyses might be useful inputs into group/policy decision making in some indirect and unspecified way [5,6]. The question remains as to whether the preferences of individual citizens, via preference-based subgroups, should have a formal, direct role in cost-effectiveness analysis and policy formation. This is particularly important in relation to resource-consuming decisions in collectively-funded public health services.

The case for acknowledging patient heterogeneity in preferences has been convincingly made by Sculpher in the context of menorrhagia therapy within the National Health Service for England and Wales [7], following the earlier work of Nease and Owens [8]. Sculpher confirmed that the two available interventions maximised the patient-specific QALYs for one subgroup of women; hence a strategy of offering treatment based on individual preferences at the point of care would, at least in principle, be a cost-effective public policy even in the collectively-funded system considered. This stimulated discussion about the possibility of implementing fully individual *patient* preference-based QALYs [9,10], a route subsequently explored by Basu and Meltzer [11-13] when developing their Expected Value of Individualised Care measure, and later by others [14-18].

However, none of these researchers seem enthusiastic about treating subgroup preferences as fundamental phenomena in driving health policy. Their implicit assumption is either that individual or subgroup preferences can be reduced to, and treated as, epiphenomena, i.e. as effectively being ‘caused’ by the biological-clinical and/or socio-demographic characteristics of the person or subgroup; or that preferences can be given policy relevance only if interpreted and processed through their associations with observable/verifiable objective characteristics of persons. The one exception, which ‘proves the rule’ - because subgrouping is not involved - is when preferences are elicited at the population level and used to produce a mean tariff applied to all individuals, as in the EQ-5D tariff used in QALY-based analyses. *If* it were decided to treat subgroup preferences as valid and independent determinants of public policy, a transparent analytical procedure will be needed.

The aim of this study is to present a procedure combining two analytical techniques that have not, thus far, featured in the debate: (i) Cluster Analysis (CA) which is used to generate preference subgroups, and (ii) Multi-Criteria Decision Analysis (MCDA) which provides the explicit policy framework for including clustered preferences. Our study has an empirical basis, and the data are from a large RCT about prostate cancer screening. However, the focus is on providing a proof of method for preference subgrouped public policy (via CA and MCDA). Thus the results are presented as a practical background to the discussion we hope to generate on this crucial issue. Our illustration highlights a number of issues that are likely to arise in any substantive implementation.

## Methods

The two techniques used in this study, Cluster Analysis (CA) and Multi-Criteria Decision Analysis (MCDA), are separately well-established. However, their combined use in health-related research, as we propose, is innovative. We could only locate one other application of the idea, in production economics, where it was used to evaluate e-commerce enterprises [19]. Before turning to these techniques, we describe the data.

### The data

For input into a public policy decision framed as a MCDA we required individual preferences from a representative sample of the population, expressed in the form of importance weights for different criteria relating to the decision. We used data from one arm of a Randomised Controlled Trial (RCT) of two online decision aids for Australian men aged 40-69 considering Prostate-specific Antigen (PSA) testing for prostate cancer, which was available and in the required format.^1^

Five criteria were provided in this arm of the trial:

LOSS OF LIFETIME: Avoid losing 5-10% of individual’s remaining life expectancy.

NEEDLESS BIOPSY: Avoid having a needless biopsy.

URINARY PROBLEMS: Avoid urinary problems after treatment for prostate cancer.

BOWEL PROBLEMS: Avoid bowel problems after treatment for prostate cancer.

SEXUAL PROBLEMS: Avoid sexual problems (impotence) after treatment for prostate cancer.

These criteria were developed in the context of an individual decision aid, but we believe they are a reasonable set to explore as the effectiveness side of a public policy issue in a proof of method.

The criteria selected were based on the findings of a General Practitioner (GP) pilot study, a full account of which has been presented [20]. GPs provided information on the criteria we had included in the earlier version of the decision aid and other factors they thought were important for patients in making a decision about PSA testing, thereby supplementing findings from the literature.

The RCT itself was based on a community sample of 1,970 men aged 40-69 years in 2011. Of these, 727 men were allocated to the arm where the interactive decision aid consisted of the five criteria outlined above.

**Figure 1:**
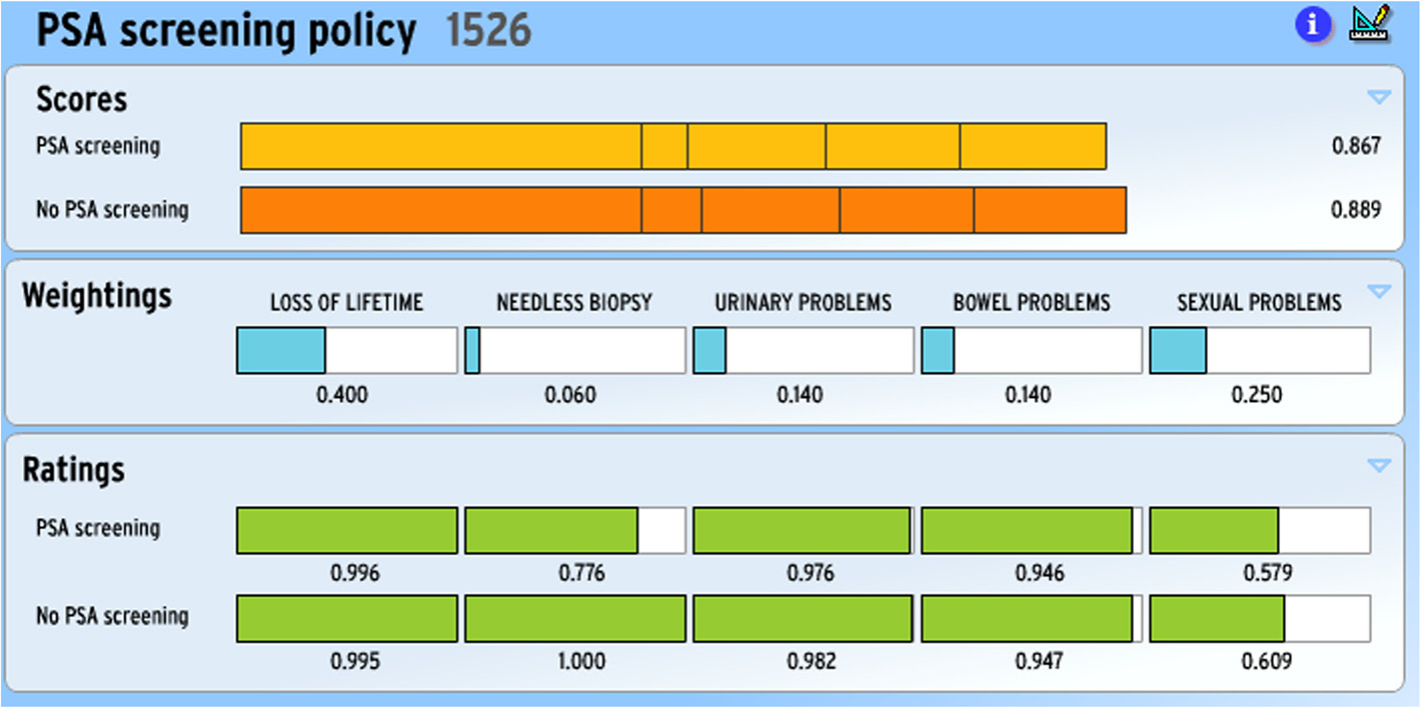
Annalisa MCDA screen with data for respondent 1526 in PSA decision aid trial.

The criterion weightings provided by respondent number 1526 can be seen in Figure 1, which displays the full MCDA decision aid screen. Using this web-based decision aid template, the importance weightings were elicited by respondents dragging the cursor to change the bar lengths, dynamically normalised to add to 100%. (MCDA as a technique does not elicit the inputs into it, but in this case the template was used as the preference-eliciting device.) The bottom Ratings panel contains the evidence base for the analysis in the form of the performance rates for the two options on the five criteria [20]. These ratings were made available to the respondent after their weightings had been elicited. (They were able to change their weightings after seeing this data, but virtually none did this and so it is the original weights which are clustered.) The top panel displays the scores for the two policy options, which result from combining the weightings of respondent number 1526 with the evidence-based ratings by way of a simple expected value calculation.

The only men excluded at survey entry were those with diagnosed prostate cancer. There were no exclusions for men ‘at risk’, so the 523 men whose preferences were cluster analysed included those reporting a first degree relative with prostate cancer (17%), or being unsure thereof (9%). 204 of the original 727 respondents had been previously excluded on the grounds that they had, at two distinct points in the survey, clicked the same point on a 10 point scale 8 times in a row as likely non-serious responders. (Respondents were recruited by an agency and received points for completion.)

The remaining 523 sets of criterion weights were analysed using CA to produce sets of subgroup means for input into MCDAs of PSA testing.

We supply the above details to give the reader some background to the importance weights being clustered, but emphasise that the methods by which they were elicited are largely irrelevant to our proof of method. Sets of weights may be produced by diverse methods, including Discrete Choice Experiments, and are suitable for clustering so long as they produce a full set of attribute weights for each individual.

### Cluster analysis

CA and its various implementations are described in many texts [21-23]. There are several implementation packages, such as the R statistical package which was used in this study [24]. CA has been widely used in subgrouping on the basis of observable characteristics, ranging from types of gut bacteria at the cellular level [25] to the human level, where it is proving useful in the definition, diagnosis, and treatment of complex conditions, such as back pain [26,27] and fibromyalgia [28]. Bass and colleagues [29] used one of the main types of CA (k-means) in pursuit of their aim of nudging Afro-Americans towards colorectal cancer screening, identifying three subgroups which they labelled ’Ready screeners’, ’Fearful avoiders’ and ‘Cautious screeners’.

### Clustering

Three different techniques of CA were employed in this study to demonstrate not only that different techniques produce different clusters, but that choosing among clustering techniques is an important task itself in implementing the approach. We used Latent Class Analysis (MCLUST), Partitioning Around Medoids (PAMK) and Hierarchical Agglomeration via Ward’s method (HCLUST), presenting the solutions generated by requesting 2, 3 and 4 clusters. The silhouette widths cluster quality indicator introduced below was calculated for solutions up to 9 clusters. The 2, 3 and 4 clusters included the maximal widths for all three methods and it was necessary to choose the same set for this comparative analysis. In all cases we used the R statistical package noted in parentheses, which makes our analyses accessible on an open source basis.

*Latent Class Analysis* (LCA) employs a model-based approach in which probabilities of cluster membership are estimated, and individuals are assigned to the cluster for which their membership probability is highest.

In *Partitioning* methods the cluster membership of an individual and hence the membership of clusters changes throughout the process. The aim is to find a solution that minimises the internal variance within clusters relative to a specified centroid (e.g. the medoid, or mean in the kmeans partitioning) and maximises the distance between cluster centroids.

In *Hierarchical Agglomeration* methods, individuals are progressively grouped in terms of their distance from each other in n-dimensions, where n is the number of criteria for clustering. Once assigned to a cluster they remain in that cluster, while the process of allocating unassigned individuals continues. The Ward’s method is a special case, which assigns individuals to minimise the internal distance of each cluster at that point in the process.

Following the clustering analyses, and testing to see if the cluster solutions provided groupings significantly different on the five criteria using ANOVA, we allocated interpretive labels for each solution based on the weight assigned to the highest weighted criterion, and assessed the quality of the clusters produced by the alternative solutions. The evaluation of cluster solutions, which involves establishing the optimal number of clusters, as well as the quality of the grouping, has been the subject of continuing research since the early papers [30,31]. These issues are summarized [32].

It is widely acknowledged that cluster quality assessment is inherently multi-dimensional. Raskutti and Leckie (1999) suggest four criteria, but two of these four - the *compactness* of the cluster (i.e. the mean intra-cluster distance of observation from the centroid) and the *isolation* of the clusters (i.e. the mean inter-cluster distance) - are the ones most commonly used. They are the basis of the silhouette coefficient measure we chose [31]. Summary measures of cluster validity, and numerical differences between clustering solutions on such measures, must be interpreted in the light of the application of the clusters [22]. Considerations of efficiency and equity may lead to selection of a clustering solution which is not highest, or even very highly rated, in terms of purely statistical quality. In marketing, numerous other criteria impact on the selection of a cluster solution. Statistical quality is only one of these. The ten criteria below, collated from the marketing area [33], are all *potentially* relevant in our case. We would omit only criterion two, given our belief that preferences should be elicited directly and separately from ‘objective’ characteristics, in order not to treat people as a bundle of characteristics. We have translated the marketing terms into ones more appropriate for a health service setting:

1. Substantial: The subgroups are large enough to serve efficiently.
2. Accessible: The subgroups can be effectively reached and served, which requires them to be characterized by means of observable variables.
3. Differentiable: The subgroups can be distinguished conceptually and respond differently to different policy-mix elements and programs.
4. Actionable: Effective programs can be formulated to attract and serve the subgroups.
5. Stable: Only subgroups that are stable over time can provide the necessary grounds for a successful strategy.
6. Parsimonious: To be administratively meaningful, only a small set of substantial clusters should be identified.
7. Familiar: To ensure political acceptance, the subgroups composition should be comprehensible.
8. Relevant: Subgroups should be relevant in respect of the service’s competencies and objectives.
9. Compactness: Subgroups exhibit a high degree of within-subgroup homogeneity and between-subgroup heterogeneity.
10. Compatibility: Subgroup results meet other administrative requirements.

Applying such criteria in a substantive application of our method is a task for which we believe MCDA is appropriate since it provides increased transparency in terms of specification of the importance attached to each criterion (the weightings) and the performance ratings of the available options on the criteria, as well as an explicit algorithm for combining the ratings and weightings to produce an overall opinion (the scores). Selecting a set of criteria and assigning importance weightings to them is one part of the task approached in this way. Arriving at ratings for how well each clustering technique/solution performs on each of the selected criteria is the second task. Integrating the weightings and ratings into an overall evaluation of each option is the final requirement, and in MCDA this is normally done using the expected value principle.

We fully accept that whether or not MCDA is the best, or an appropriate, approach to this task is itself a multi-criterial decision, involving both performance ratings and preferences.

### Multi-criteria decision analysis

MCDA and its various forms are described and surveyed in numerous texts [34-39] and there are many examples of its use [1,37,40-45]. A large number of software implementations exist, reflecting both varying versions of MCDA and judgements about the extent and type of complexity to be catered for, as well as the time and cognitive resources required [46-49]. In the illustrative analyses reported here we employ Annalisa©, as used in the trial. Annalisa is an implementation of the simple linear additive version of MCDA, in which the scores for each option are produced by multiplying the performance rates for the option on each of the criteria by the respondent’s weights for those criteria, and summing across criteria. Its one-screen-fits-all interface was specifically developed to be less complex in both development and delivery than the alternatives [20,49]. However, the selection of a software implementation of MCDA, like the selection of the CA technique (and indeed software for implementing it), is not something we wish to address on the present occasion. It would be a crucial part of the policy-specific development process.

The basic Annalisa screen (Figure 1) shows the expected value Scores which result from combining the evidenced-based Ratings for each policy Option on each criteria with the respondent’s relative importance Weightings for the criteria. The data are for respondent number 1526 in the PSA trial from which our data are drawn - see below. (The No PSA score is higher for him, reflecting the importance Weightings he gave.)

### Translation into MCDA-based policy analysis

The results for each of the four cluster solutions within the three CA techniques were fed into this MCDA tool, and the subgroup scores for each policy calculated. Subsequently, we conducted sensitivity analysis in relation to the Loss of Lifetime criterion, to see what change in the percentage rating for PSA vs. No PSA screening policy would be needed to bring each subgroup into equipoise, i.e. have equal scores for the two policy options. This seemed the most interesting of the many possible sensitivity analyses to undertake from a policy perspective, given it indicates the subgroup’s trade-offs of harms with what is conventionally seen as the main potential benefit (Loss of Lifetime).

## Results

### Clustering

The clustering solutions from the three cluster techniques are shown in Table 1.^2^ The mean subgroup weightings on the five criteria relevant to the PSA test decision (Loss of Lifetime, Needless Biopsy, Urinary Problems, Bowel Problems, and Sexual Problems) are shown for each solution.

**Table 1:** **Mean cluster weights from 2, 3 and 4 cluster solutions using LCA, PAM and Ward methods**

						**MEAN CRITERION WEGHTS**					
**Clustering Method**	**Cluster Solution**	**Cluster Number**	**N (of 523)**	**%**	**Quality**	**LOSS OF LIFETIME**	**NEEDLESS BIOPSY**	**URINARY PROBLEMS**	**BOWEL PROBLEMS**	**SEXUAL PROBLEMS**	**Interpretive Label**
**Latent Class Analysis (MCLUST)**											
	4	1	327	62.5	0.24	0.22	0.15	0.20	0.20	0.23	Equals
		2	53	10.1	0.64	0.88	0.02	0.04	0.03	0.03	Very High Lifers
		3	121	23.1	0.31	0.53	0.06	0.14	0.15	0.12	Moderate Lifers
		4	22	4.2	0.39	0.13	0.53	0.11	0.11	0.12	Moderate Biopsers
					**0.31**						
	3	1	407	77.8	0.29	0.27	0.13	0.19	0.19	0.21	Equals
		2	92	17.6	0.60	0.78	0.03	0.07	0.06	0.06	Very High Lifers
		3	24	4.6	0.36	0.16	0.52	0.10	0.11	0.11	Moderate Biopsers
					**0.35**						
	2	1	493	94.3	0.25	0.36	0.11	0.17	0.17	0.18	Moderate Lifers
		2	30	5.7	0.36	0.22	0.49	0.10	0.10	0.10	Moderate Biopsers
					**0.26**						
**Partitioning Around Medoids (pamk)**											
	4	1	270	51.6	0.33	0.19	0.18	0.22	0.22	0.19	Equals
		2	59	11.3	0.63	0.87	0.03	0.04	0.03	0.03	Very High Lifers
		3	163	31.2	0.26	0.49	0.11	0.14	0.14	0.13	Moderate Lifers
		4	31	5.9	0.36	0.06	0.06	0.11	0.13	0.64	Very High Sexers
					**0.35**						
	3	1	301	57.6	0.27	0.18	0.17	0.21	0.21	0.24	Equals
		2	59	11.3	0.63	0.87	0.03	0.04	0.03	0.03	Very High Lifers
		3	163	31.2	0.30	0.49	0.11	0.14	0.14	0.13	Moderate Lifers
					**0.32**						
	2	1	346	66.2	0.40	0.21	0.16	0.20	0.20	0.23	Equals
		2	177	33.8	0.41	0.64	0.08	0.09	0.10	0.09	Very High Lifers
					**0.41**						
**Ward’s Hierarchical (HCLUST)**											
	4	1	170	32.5	0.34	0.14	0.21	0.23	0.23	0.18	Equals
		2	38	7.3	0.27	0.08	0.07	0.12	0.14	0.59	Very High Sexers
		3	60	11.5	0.68	0.86	0.03	0.04	0.04	0.03	Very High Lifers
		4	255	48.8	0.17	0.42	0.12	0.15	0.16	0.15	Moderate Lifers
					**0.29**						
	3	1	208	39.8	0.22	0.13	0.19	0.21	0.21	0.26	Equals
		2	60	11.5	0.68	0.86	0.03	0.04	0.04	0.03	Very High Lifers
		3	255	48.8	0.23	0.42	0.12	0.15	0.16	0.15	Moderate Lifers
					**0.28**						
	2	1	463	88.5	0.40	0.29	0.15	0.18	0.18	0.20	Moderate Lifers
		2	60	11.5	0.76	0.86	0.03	0.04	0.04	0.03	Very High Lifers
					**0.44**						

Also shown are cluster sizes and statistical quality (as measured by average silhouette width). The bold numbers indicate the statistical quality of the cluster solution. N.B. ANOVA showed all clusters to be significant at p < 0.05, except LCA 4/4 (Moderate Biopsers).

Differences in the clusters produced, given the fixed criterion framing of the elicitation, are apparent. However, it is also clear that 3 broad preference patterns are common to all three of the 4 cluster solutions, which are the ones we focus on henceforth:

1. A relatively small subgroup of 10-11% ‘Very High Lifers’, for whom Loss of Lifetime is almost all-important with this criterion given 86-88% weight;
2. A relatively large subgroup of ‘Moderate Lifers’, comprising 23-49% of the sample who give this criterion 42-53% weight (and hence include respondent 1526 in Figure 1);
3. The largest group of all (‘Equals’) at 33-63% of the sample, who gave roughly equal weights to the five criteria (including 14-22% weight to Loss of Lifetime).

Setting these three subgroups apart, leaves a ‘Very High Sexers’ group at 7% and 11% of the sample who assigned 64% and 59% weights to the Sexual Problems criterion in the PAM and Ward solutions, respectively. They are replaced by ‘Moderate Biopsers’ at 4% with 53% weight assigned to Needless Biopsy in the LCA solution.

On the basis of roughly averaging this data, a policy based purely on Loss of Lifetime minimisation might just attract majority support.

The statistical quality of the solutions, as approximated by silhouette width, varies from .26 to .44 (see Table 1). A much reproduced scale would attach the label ‘The structure is weak and could be artificial’ to results in the .26-.5 range, but we can find no validation of this scale. In any case we believe that, as made clear earlier, clustering solutions should be evaluated by their external real-world consequences, as well as their internal qualities.

We have confirmed that different techniques and solutions produce different clusters. But also, that the resulting clusters are all capable of meaningful interpretations based on the most prominent criterion (or lack of one). However, to reiterate, we explicitly take no position on the issue of the most appropriate clustering technique, since this should be part of the policy development process and reflect the application of criteria other than statistical quality.

### Entering cluster weights into MCDAs

Pursuing our proof of method, the results from the 4 cluster solutions from the three techniques were now inserted into MCDAs.

None of the preference-based subgroups produced by *any* clustering solution favours a PSA screening policy. There are various ways in which the complex set of results could be displayed, but we feel it most informative to present just one type of sensitivity/threshold analysis. Given the *weight* assigned by a subgroup to the Loss of Lifetime criterion, what proportionate change in the *ratings* for the two policy options on this criterion would result in this subgroup being in policy equipoise (i.e. the option scores being equal in its MCDA)?

The answers for all three of the 4 cluster solutions are presented in Table 2, with Additional file 1: Tables S1, S2 and S3 providing the full calculations, and S4 an illustration of the calculation procedure.

**Table 2:** **Percentage increase in gap between relative Loss of Lifetime performance ratings for PSA and No PSA screening options needed to produce equipoise for each 4 cluster solution**

**Cluster**	**LCA**	**PAM**	**Ward’s**
**Equals**	19	25	39
**Very High Lifers**	1	1	1
**Moderate Lifers**	3	6	8
**Very High Sexers**	…	56	43
**Moderate Biopsers**	95	…	…

The table confirms that the required changes are a direct reflection of the subgroups' weights, with (in the Ward solution), Very High Lifers (86% weight to Loss of Lifetime) requiring a 1% improvement, and Moderate Lifers (42% weight) an 8% improvement. The high (39%) requirement for Equals reflects their low (14%) weight for Loss of Lifetime, which is not much greater than that of Very High Sexers. The requirement patterns in the LCA and PAM solutions are similar. But the result for Moderate Biopsers in LCA (95%) while it is consistent with the 13% weight assigned to Lifetime Loss, is a useful warning of the need to be cautious in selecting a solution. It is from the one cluster that was not significant in ANOVA (see Table 1 caption).

### Age-stratified results

Following the exclusion of those participants ‘at risk’ of prostate cancer or ‘unsure’ about their family history, the sample for age-stratified clustering became 388. 156 were in their 40s, 135 in their 50s, and 97 in their 60s.

The same type of interpretable subgroups reappear with different distributions (Additional file 1: Tables S5, S6, S7), but with notably different thresholds on the Loss of Lifetime criterion to produce equipoise. (Table 3) (These were calculated in the same way as illustrated in Additional file 1: Table S4.)

**Table 3:** **Percentage increase in gap between relative Loss of Lifetime performance ratings for PSA and No PSA screening options needed to produce equipoise for each 4 cluster solution, by age group**

	**40-49 years**		**50-59 years**		**60-69 years**	
	**% Change**	**%N**	**% Change**	**%N**	**% Change**	**%N**
**Moderate Lifers**	0.1	35	2.7	27	4.1	26
**Very High Lifers**	0.0	25	0.3	24	0.4	14
**Equals**	0.4	32	3.5	41	21.5	44
**Very High Sexers**	0.2	8	14.4	8	…	…
**Moderate Biopsers**	…	…	…	…	45.4	15

It seems a reasonable inference that age effects exist. The proportions (%N) of both Moderate and Very High Lifers increase progressively from younger to older at the same time, as their equipoise requirement progressively increases. This necessitates that the opposite happens for the proportions of the other subgroups, and we indeed observe that Equals increase from 32% to 44% moving from youngest to oldest groups. Their equipoise requirement also rises dramatically, from near equipoise for the 40s (0.4%) to 21.5% for the 60s. The residual subgroup proportion increases from 8 % to 15%. In the 40s and 50s it is the Very High Sexers, who are in virtual equipoise in the 40s, but significantly divergent from it in the 50s (14.4% requirement). However, in the 60s this subgroup is replaced by Moderate Biopsers, a cluster dominated by concern with needless testing.

All these variations have modest appeal in terms of face validity, but any inferences need to be drawn with caution, since the three clustering solutions are for different datasets (albeit from same responders), and so are not directly comparable. These age effects are the combined effect of different criterion performance ratings for the age groups as well as different preference patterns.

## Discussion

This study presents an example of how public preferences *could* be incorporated into policy decisions respecting both the multi-criterial nature of those decisions and the heterogeneity of the population in relation to their weightings. The various methodological and practical issues to be addressed in implementing such an approach are emphasised. Always to be determined are: the structure of the policy decision (options, criteria in the MCDA); the choice of MCDA version and implementation software; the choice of CA technique; the choice of number of cluster solutions and measure of cluster quality; and the trade-offs between statistical quality and other criteria. It is the primary aim of this paper to ensure that these issues are addressed transparently, rather than dealt with in an exclusively deliberative process.

Objections to cluster analysis as an ‘unsupervised’ technique only to be used in abductive hypothesis generating – with the resulting clusters requiring ‘validation’ against some other criterion and insertion into a hypothesis testing framework [27] – are of little relevance to our approach. There is no gold standard against which preference clusters can be compared. We have made clear that regression of preference clusters on biological-clinical or socio-demographic variables is inappropriate, because we are in a policy/decision making practice context, not a hypothesis-testing or scientific research-driven one.

While the decision on which solution to adopt in the presence of clustering differences requires consideration of factors other than statistical quality, one thing should not enter into analysis at the policy level in relation to preference subgrouping regardless of the method used: the characteristics of those individuals who move between clusters depending on the technique and solution. Tracing such individual movements is feasible in all software implementations of cluster analysis, but there seems to be no conceptual justification for doing so. In this sort of analysis an individual is simply a person expressing their preferences in the context of a particular decision. It is vital they are not treated as a ‘bundle of variables’. In some practice contexts it will be appropriate to explore the statistical relationship between preference-based subgroups and objective characteristics, typically via regression analysis. Or to look forward and explore the relationship with some future outcome or behavior, probably also via regression analysis. But we argue that neither of these explorations is appropriate when it involves reducing the preferences of a person, or group of persons, to a set of predictive or predictor variables, since this undermines the fundamental personhood of the preference-bearer [50].

A mini-debate provoked by a comment by Robinson and Parkin on their paper [51,52] made clear that one central issue is whether public or patient preferences are appropriate. We are explicitly operating in the extra-welfarist framework where *stated public preferences over outcomes* are the inputs relevant for a subgrouped public policy, not *revealed patient choice of options*. In a collectively-funded health care system we take the view that it is the preferences of members of the public, as citizens which are the appropriate inputs into policy, leaving patient preferences to be applied at the individual/clinical level within the constraints set by community policy. Of course, there is nothing in the techniques themselves which rule out using patient preferences as inputs, but the conflict of personal and public interest at, or near, the point of care, poses major challenges to using those of patients.

We do not address the cost side of policy making here, instead concentrating on how subgroup preferences in relation to effectiveness criteria could be incorporated into Cost-effectiveness Analysis and public policies. As emphasised by Claxton it is important that an MCDA-based policy operating within a budget constraint respects the existence of opportunity costs, ensuring that any net benefit foregone from the expansion of the criteria on the effectiveness side (beyond QALYs) should be taken into account [53].

In an extended MCDA framework it would be possible to include options that fall within of the South-West quadrant of the cost-effectiveness plane, i.e. are cost-effective by being less effective, but proportionately much cheaper, than the standard one [54]. And one might include an explicit ‘Net effect on (generalised) others’ criterion for individual respondents to *weight*. In the extreme, this could be split into two on the basis of the ‘just deserts’ criteria that emerges in most public surveys. We are not advocating this, simply confirming that moving to an MCDA-based public policy will make such issues and their resolution more transparent.

A crucial finding in the Raskutti and Leckie paper, replicating that of Macskassy, is that humans asked to cluster the same data as a CA program, produce equivalent variation in both the optimal number of clusters and their content [32,55]. In other words, individual policy makers engaging in subgrouping are unlikely to outperform a cluster solution, so the same discussion will be needed if policy makers undertake the task.

## Conclusions

In attempting to respect the heterogeneity of population preferences in public policy, a subgroup approach of some sort is inevitable. In this paper we illustrate how two types of analysis might, in combination, represent a viable approach. The implementation of Cluster Analysis and Multi-Criteria Decision Analysis, individually and in combination, poses major challenges - conceptual, methodological, ethical-political, and practical. We outline these challenges in the paper, stressing that most are only exposed by these more analytical techniques, not created by them. Alternative analytical or deliberative approaches will face similar challenges, and any proper evaluation must involve comparison of the approaches in empirical practice, not simply against diverse sets of normative principles. This is particularly important because computer technologies quickly expose the ‘digital divide’, easily obscured in deliberative approaches. Such unbiased comparative evaluation is the next item on the research agenda.

The empirical results from our PSA screening example are consistent with the trend away from advocacy of PSA screening of asymptomatic men without a family history of prostate cancer, based on both worries about the test and preference considerations [56]. But the fact that our results are in line with this observed trend should not be misinterpreted. All we have sought to show as proof of method, is that one can carry out analyses that identify the improvement in criterion performance (e.g. a superior test, less subsequent problems from treatment) needed for a preference-based subgroup to favour a screening policy.

Our finding of age-based preference subgrouping raises the question of whether sub-subgrouping individual preferences on bases such as age, sex, ethnicity, or religion is consistent with truly *person*-centred public policy.

## Endnotes

^1^ The trial from which the data come was approved by the University of Sydney HREC (Protocol No.: 05-2011/13712) on May 13 2011 and was included in the Australian New Zealand Clinical Trials Registry (ANZCTR) on 6 July 2012 (ACTRN12612000723886) (https://www.anzctr.org.au/Trial/Registration/TrialReview.aspx?id=343044).

^2^ An early version of this paper was presented in a poster at the Lancet Public Health Science conference in November 2013 [57]. This contains links which will enable the reader to engage in interactive exploration of the data in a downloadable spreadsheet and to explore the survey as seen by a respondent.

## Additional file

Additional file 1: Table S1. LCA 4 cluster solution subgroup mean weights input into MCDA, policy scores generated and threshold on Loss of Lifetime identified. **Table S2.** PAM 4 cluster solution subgroup mean weights input into MCDA, policy scores generated and threshold on Loss of Lifetime identified. **Table S3.** Ward 4 cluster solution subgroup mean weights input into MCDA, policy scores generated and threshold on Loss of Lifetime identified. **Table S4**. Derivation of proportionate change in Loss of Lifetime ratings for the policy options required by Very High Sexers subgroup (Ward 4 solution) to achieve policy equipoise. **Table S5.** Ward 4 cluster solution subgroup mean weights for 40-49 year olds input into MCDA, policy scores generated and threshold on Loss of Lifetime identified. **Table S6.** Ward 4 cluster solution subgroup mean weights for 50-59 year olds input into MCDA, policy scores generated and threshold on Loss of Lifetime identified. **Table S7.** Ward 4 cluster solution subgroup mean weights for 60-69 year olds input into MCDA, policy scores generated and threshold on Loss of Lifetime identified.

## Abbreviations

ANOVA: Analysis of Variance
CA: Cluster Analysis
GP: General Practitioner
LCA: Latent Class Analysis
MCDA: Multi-Criteria Decision Analysis
NICE: National Institute for Health and Care Excellence
PAM: Partitioning Around Medoids
PSA: Prostate-Specific Antigen
QALY: Quality-Adjusted Life Year
RCT: Randomised Controlled Trial
