# Supplementary material for: Addressing preference heterogeneity in public health policy by combining Cluster Analysis and Multi-Criteria Decision Analysis: Proof of Method
Source: Health Econ Rev. 2015 May 14;5:10. doi: 10.1186/s13561-015-0048-4 (PMC4429422; doi:10.1186/s13561-015-0048-4)
Supplement: Additional file 1: Table S1. — LCA 4 cluster solution subgroup mean weights input into MCDA, policy scores generated and threshold on Loss of Lifetime identified. Table S2. PAM 4 cluster solution subgroup mean weights input into MCDA, policy scores generated and threshold on Loss of Lifetime identified. Table S3. Ward 4 cluster solution subgroup mean weights input into MCDA, policy scores generated and threshold on Loss of Lifetime identified. Table S4. Derivation of proportionate change in Loss of Lifetime ratings for the policy options required by Very High Sexers subgroup (Ward 4 solution) to achieve policy equipoise. Table S5. Ward 4 cluster solution subgroup mean weights for 40-49 year olds input into MCDA, policy scores generated and threshold on Loss of Lifetime identified. Table S6. Ward 4 cluster solution subgroup mean weights for 50-59 year olds input into MCDA, policy scores generated and threshold on Loss of Lifetime identified. Table S7. Ward 4 cluster solution subgroup mean weights for 60-69 year olds input into MCDA, policy scores generated and threshold on Loss of Lifetime identified. [file 13561_2015_48_MOESM1_ESM.docx]

**Supplementary Tables:**

Additional file 1: Table S1

Additional file 1: Table S1: LCA 4 cluster solution subgroup mean weights input into MCDA, policy scores generated and threshold on Loss of Lifetime identified

Additional file 1: Table S2

Additional file 1: Table S2: PAM 4 cluster solution subgroup mean weights input into MCDA, policy scores generated and threshold on Loss of Lifetime identified

Additional file 1: Table S3

Additional file 1: Table S3: Ward 4 cluster solution subgroup mean weights input into MCDA, policy scores generated and threshold on Loss of Lifetime identified

Additional file 1: Table S4

Additional file 1: Table S4: Derivation of proportionate change in Loss of Lifetime ratings for the policy options required by Very High Sexers subgroup (Ward 4 solution) to achieve policy equipoise.

Section A contains the subgroup's mean criterion weightings, including their 8% for the Loss of Lifetime criterion. In section B the performance ratings for the two options on the five criteria appear. Section C presents the expected value score for each option that result from multiplying these weightings by the ratings for the option and summing. It also presents the gap between the two scores (.034) resulting from subtracting the PSA score, which is always the lower, from the No PSA one. Finally, section D shows (as ‘SGCW’) the absolute reduction in the No PSA rating (.4291) that would be necessary for the subgroup scores to be equal. 'Threshold' identifies, by subtraction from PSA rating, the required No PSA rating (.5669) and '%Req' expresses the required absolute reduction as a proportion of the PSA rating.

Additional file 1: Table S5

Additional file 1: Table S5: Ward 4 cluster solution subgroup mean weights for 40-49 year olds input into MCDA, policy scores generated and threshold on Loss of Lifetime identified.

Additional file 1: Table S6

Additional file 1: Table S6: Ward 4 cluster solution subgroup mean weights for 50-59 year olds input into MCDA, policy scores generated and threshold on Loss of Lifetime identified

Additional file 1: Table S7

Additional file 1: Table S7: Ward 4 cluster solution subgroup mean weights for 60-69 year olds input into MCDA, policy scores generated and threshold on Loss of Lifetime identified.
